# Utilizing GCaMP transgenic mice to monitor endogenous G_q/11_-coupled receptors

**DOI:** 10.3389/fphar.2015.00042

**Published:** 2015-03-09

**Authors:** John G. Partridge

**Affiliations:** ^1^Department of Pharmacology and Physiology, Georgetown University School of Medicine, Washington, DC, USA; ^2^Interdisciplinary Program in Neuroscience, Georgetown University School of Medicine, Washington, DC, USA

**Keywords:** GCaMP, Cre-loxP, G protein-coupled receptor, Ca^2+^ measurement

## Abstract

The family of GCaMPs are engineered proteins that contain Ca^2+^ binding motifs within a circularly permutated variant of the *Aequorea Victoria* green fluorescent protein (cp-GFP). The rapidly advancing field of utilizing GCaMP reporter constructs represents a major step forward in our ability to monitor intracellular Ca^2+^ dynamics. With the use of these genetically encoded Ca^2+^ sensors, investigators have studied activation of endogenous G_q_ types of G protein-coupled receptors (GPCRs) and subsequent rises in intracellular calcium. Escalations in intracellular Ca^2+^ from GPCR activation can be faithfully monitored in space and time as an increase in fluorescent emission from these proteins. Further, transgenic mice are now commercially available that express GCaMPs in a Cre recombinase dependent fashion. These GCaMP reporter mice can be bred to distinct Cre recombinase driver mice to direct expression of this sensor in unique populations of cells. Concerning the central nervous system (CNS), sources of calcium influx, including those arising from G_q_ activation can be observed in targeted cell types like neurons or astrocytes. This powerful genetic method allows simultaneous monitoring of the activity of dozens of cells upon activation of endogenous G_q_-coupled GPCRs. Therefore, in combination with pharmacological tools, this strategy of monitoring GPCR activation is amenable to analysis of orthosteric and allosteric ligands of G_q_-coupled receptors in their endogenous environments.

## INTRODUCTION

Guanosine nucleotide-binding proteins (G proteins) are intracellular proteins involved in transmitting signals from outside a cell to the inside of the cell (Oldham and Hamm, 2008). Since their initial detection in the 1960’s by Nobel laureates Martin Rodbell and Alfred Gilman, heterotrimeric forms of G proteins (G_α_, G_β_ and G_γ_) have received much consideration in the general fields of pharmacology, biochemistry and neuroscience. This is justified as their interacting, coupled receptors have been an established source of clinically active therapeutics. Several G proteins contain lipid modifications on one or more of their subunits to enable targeting to the plasma membrane while facilitating protein interactions. The precise arrangement and targeting of subunits in heterotrimeric G proteins affects not only which receptor with which it can interact, but also the downstream effector target. This general scheme of extracellular signal transduction has been selected for across evolution and has been repeated in nature abundantly. Built-in flexibilities originating from unique receptors, G-proteins and effectors provide the means to distribute distinct physiological response pathways to external stimuli ranging from photons to complex protein hormones (Katritch et al., 2013).

There are ∼16 genes found in human that encode different forms of G_α_ which belong to a larger group of enzymes called GTPases. The G_α_ subunit of heterotrimeic G proteins is highly controlled by factors that influence its ability to bind to guanosine triphosphate (GTP). The GTPase activity of G_α_ proteins hydrolyze GTP to guanosine diphosphate (GDP). When bound by GTP, G_α_ is considered in an active state and when bound by GDP, G_α_ is in a less active state (Limbird, 2004). The time course of the G protein signal is controlled by the duration of the GTP-bound alpha subunit, which can be regulated by RGS (regulators of G protein signaling) proteins, GEFs (guanine nucleotide exchange factors) or by covalent modifications. G_α_ subunits mediate the signal transduction pathway that initiates from an agonist occupied receptor to numerous intracellular effector proteins. For example, G_α_ subunits in the G_α_ family stimulate the production of 3′-5′-cyclic adenosine monophosphate (cAMP) by activation of adenylyl cyclase. Another prominent branch of this family of biological signaling tools includes G_αq_.

**FIGURE 1:**
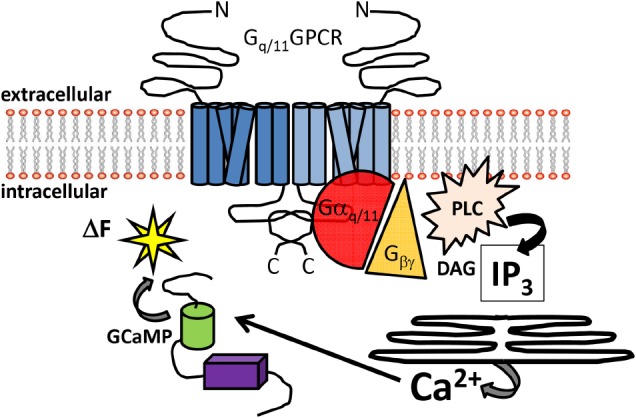
**Overview of G_q/11_-coupled GPCR signal detection by GCaMP sensors.** Schematic diagram illustrating the canonical signaling pathway of G_q/11_-coupled GPCRs. Heptahelical dimeric receptors bound by agonist can activate the G_q/11_ pathway to increase intracellular Ca^2+^. The signaling intermediaries facilitating Ca^2+^ elevations include generation of IP_3_ and DAG from PLC activation, gating of IP_3_ receptors embedded in the ER and release of intracellular Ca^2+^ stores. Free Ca^2+^ can then bind to GCaMP sensors in the cytoplasm, resulting in enhanced fluorescence. Abbreviations: N: amino-terminus C: carboxy-terminus; PLC: phospholipase C; DAG: diacylglycerol, IP_3_: inositol trisphosphate; ΔF: change in fluorescence.

G_αq_ and a closely related gene G_α11_, are broadly expressed and maintain homeostatic processes in digestive, urinary, cardiovascular and central nervous systems (CNS). It is critical to appreciate that activated G_q/11_ results in several parallel signaling pathways that include mitogen activated protein kinase (MAPK) and the phosphatidylinositol-3-kinase (PI3K)/AKT pathways. However, the pathway in which GTP bound G_q/11_ (as well as some combinations of G_βγ_) can stimulate the activity of the effector protein phospholipase Cβ (PLCβ) is the most studied (Wettschureck and Offermanns, 2005). PLCβ hydrolyzes phosphatidylinositol 4,5-bisphosphate (PIP_2_) to diacyl glycerol (DAG) and inositol trisphosphate (IP_3_). An increase in intracellular IP_3_ rapidly gates ionotropic IP_3_ receptors embedded in membranes of endoplasmic reticulum (ER). IP_3_ receptor activation results in the flux of calcium ions (Ca^2+^) from highly concentrated internal ER stores to low concentration intracellular cytoplasmic regions via channel gating (Figure 1). It is this branch of the canonical G_αq/11_ signal transduction pathway that will be the focal point of this review.

A vast amount of research has recognized an enormous array of extracellular and intracellular stimuli that dictate changes in the intracellular second messenger Ca^2+^. The concentration over time profile of this divalent cation has variable functions in nearly every cell type throughout the animal kingdom (Berridge, 2006). Cells devote considerable energy in adjusting and maintaining a steep gradient between intracellular (<1 uM) and extracellular (>1 mM) Ca^2+^ concentrations. Intracellular calcium signals regulate processes that operate over time ranges varying from milliseconds to days. One general class of calcium mobilizing external stimuli includes agonists acting at G_q/11_-coupled receptors. Examples of some G protein-coupled receptors (GPCRs) that preferentially interact with G_q/11_ include the group I metabotropic glutamate; M1, M3, and M5 muscarinic acetylcholine; 5-HT_2_ serotonergic, α1 adrenergic, vasopressin, angiotensin II and histamine H1 receptors among several others.

A great deal of our knowledge base of these receptor subtypes has its origins in the cloning era of these genes. The coding regions of many G_q/11_-coupled GPCRs were inserted into recombinant, mammalian directed expression vectors and subsequently driven to be transcribed by strong promotors into a variety of host cells. Some of these expression studies have greatly contributed to our atomic level structural understanding and knowledge of these critical receptor subtypes. Expression strategies have served many other useful purposes including pharmacological profiling, detailed determination of signal transduction pathways as well as site-directed mutagenesis studies of critical amino acids involved in structure, function and ligand binding. However, one caveat with this general paradigm is the issue of over-expression of the receptor.

Does placing too many of the same receptor type bias a signal transduction pathway through mass action relationships? Can over-expression lead to too many spare receptors and lead to aberrant constitutive activity? Questions like these have been addressed and will need to be monitored heading into the future utilizing this set of critical tools and methodologies. Nevertheless, methods examining G_αq/11_-coupled GPCRs in their endogenous states, which more closely reflect the natural environment, are becoming sharper and increasingly more available. The focus of this review article will address combining methods and paradigms from the fields of calcium imaging, mouse genetics and pharmacology to uncover endogenous G_αq/11_-coupled GPCRs and their responses to acute or sustained stimulation at the molecular and cellular levels.

## GENETIC IDENTIFICATION OF CELLS WITH ENDOGENOUS GPCRs

**FIGURE 2:**
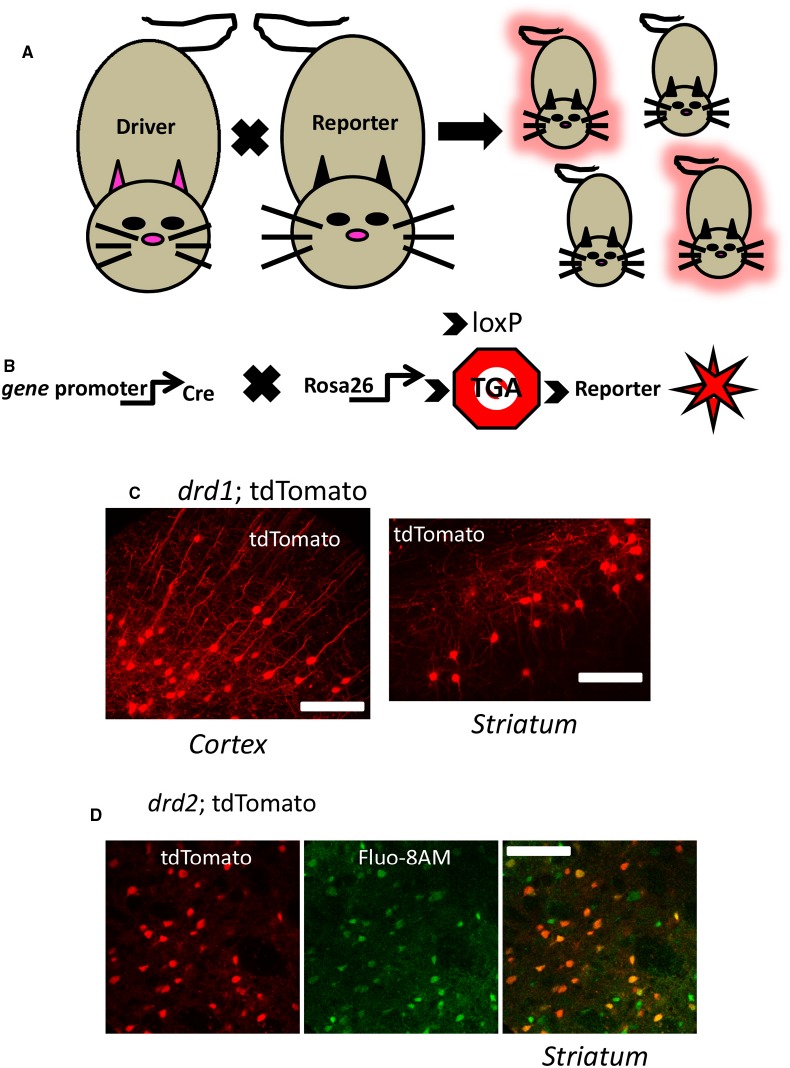
**Genetic identification of cells using cre-lox driver: reporter methods. (A)** Mating a gene promotor containing “*driver*” mouse directed to express cre in a selective fashion to a cre-dependent “*reporter*” mouse yields offspring which may inherit the driver-reporter combination (*red* offspring) of genes. Those offspring which do not inherit the combination will not express the identification marker (*non-fluorescent* offspring). **(B)** A currently used strategy is to cross a cre driver mouse to a mouse expressing a cre-sensitive element at a ubiquitously expressed locus like *rosa26*. At this locus is a Stop codon (*Stop sign with TGA*) flanked by loxP sites (indicated by *arrows*) designed to be excised by cre recombinase activity allowing expression of genetically encoded fluorescent reporters (*red star* symbol) in the target cell population. **(C)** Illustrates a confocal image of a coronal mouse brain section from an animal inheriting the *drd1*; cre and *rosa26*; tdTomato genes described above, permitting fluorescent detection of dopamine D1 cells in cortex (*left*) and striatum (*right*). **(D)** Live confocal image of an acute striatal brain slice showing *drd2*: tdTomato neurons (red, *left*) and the same section bulk loaded with the calcium sensitive dye Flou-8 (green, *center*), allowing genetic identification of striatal neurons while imaging calcium (superimposed images, *right*). See Partridge et al. (2014) for methods. Scale bars in **(C)** and **(D)**: 100 μm.

Investigations into the role of endogenous G_q/11_-coupled GPCRs in selective cells has become more prevalent using the power of transgenic animals. Temporally and spatially regulated genes can be monitored with fluorescent microscopy in mice by utilizing a DNA recombination system based on Cre recombinase. Cre based systems using P1 bacteriophage Cre recombinase which catalyze the excision of DNA located between flanking loxP sites, has been widely used since its first application in eukaryotic cells and transgenic mice (Sauer, 1987; Novak et al., 2000). Because recombination does not occur between the loxP sites until Cre is introduced, the modifications are termed conditional alterations. It is a conditional situation based on where and when the Cre recombinase gene is expressed. This powerful strategy permits the design of mouse lines with silent genetic manipulations (i.e., the flanking loxP sites) that can be un-silenced by Cre mediated recombination. In the beginning years of the twenty-first century, it became common in many laboratories to breed unique Cre recombinase “driver” mice to a mate carrying a silent flanking loxP mutation. Offspring could be produced that eliminated a coding region of a gene to generate tissue selective knock-out of a gene of interest. More recently, a variant of this general strategy has become popular by placing stop codons in between the loxP sites so as to “report” a gene, rather than eliminate it (Madisen et al., 2010). The usefulness of this technique is enhanced with distinct, commercially available reporter lines of mice (Figure 2). Illustrated in Figure 2 is an example of this latter method that permits fluorescent identification of target cells or tissues that may express a G_q/11_-coupled GPCR like neurons in layer five of cortex or striatal projection neurons.

A more straightforward genetic alternative to this approach includes the use of mice carrying bacterial artificial chromosome (BAC) transgenes. It is now routine to accurately drive the expression of genetically programmed fluorescent reporters (e.g., eGFP or tdTomato) or Cre recombinase in specific cellular populations with these large (150–350 kbp) transgenes. The GENSAT (Gene Expression Nervous System Atlas) project has used this technology to generate mouse lines with targeted cellular expression of eGFP or Cre recombinase (Gerfen et al., 2013). One advantage that the BAC insertion technology has over earlier transgenic methods is that the longer expanse of DNA encompasses much more non-coding regions containing information to direct the accurate expression of the reporter gene in time and space.

One critical assumption in using these methods is that the transgene insertion does not affect the normal physiology of an animal. However, the largely random chromosomal integration site of the BAC construct could have aberrant side effects on standard gene expression. Further, the large size of the insertion could contain unknown regulatory elements of other genes, again resulting in disruption of native genes. It is generally assumed that these are low probability events. However, in one important example in the recent literature, Kramer et al. (2011) described that Swiss Webster (SW) mice, homozygous for the *drd2*-eGFP BAC transgene had an altered phenotype (Kramer et al., 2011). However, subsequent manuscripts described the use of alternative background strains and/or reducing the copy number of BAC insertions to help control for possible affects that could lead to misinterpretations of data (Chan et al., 2012; Nelson et al., 2012). Together, all of these studies imply that BAC transgenic mice are extremely valuable tools that can be utilized to advance our understanding of endogenous GPCRs in defined cells. However, the data that results from these animals should be interpreted with the awareness of possible genetic misregulating elements contained in the BAC construct themselves or due to insertion site disruption of native genes.

## DETECTION OF CALCIUM IONS WITH DYES OR “GCaMP” VARIANTS

As stated above, changes in intracellular Ca^2+^ ([Ca^2+^]_i_) can represent a fundamental change of state in many cell types. Biological processes ranging from cardiac muscle contraction, insulin secretion, cell adhesion, proliferation or cell death represent cellular and molecular reactions dependent upon [Ca^2+^]_i_. Notably, these signals vary with a time course of milliseconds in the case of muscle contraction, to minutes in the case of sustained insulin secretion, to hours or days in some cases of programmed cell death. Therefore, it is critical that the period of time in which [Ca^2+^]_i_ changes occur, can be reliably monitored in an endogenous environment. By measuring the kinetics of calcium transients, important information can be inferred such as properties of ligand kinetics, receptor reserve and amplification of signaling (Charlton and Vauquelin, 2010).

The rich history of [Ca^2+^]_i_ detection which dates back to the 1960’s has recently been well reviewed (Grienberger and Konnerth, 2012). Briefly, bioluminescent calcium binding proteins like aequorin, or synthetic compounds like arsenazo III that changed absorbance spectrum with increasing calcium gave way to covalently modified hybrids of calcium chelating agents like EGTA or BAPTA (Shimomura et al., 1962; Brown et al., 1975; Tsien, 1980). This latter group of calcium indicator dyes, including the popular Fura-2, contains a fluorescent chromophore that can be monitored with light detection hardware. Fura-2 can be interchangeably excited with ultraviolet light at 340 nm or 380 nm in wavelength, and the ratio of the emitted light intensity at those two variable excitation wavelengths is directly correlated to the amount of intracellular calcium (Grynkiewicz et al., 1985).

Over the course of the past few decades, improvements in several variants of these fluorescent calcium indicators have been developed that exhibit an increase in fluorescence upon binding Ca^2+^. Cells can readily absorb membrane permeant acetyloxy-methyl (AM) ester forms of these compounds by adding the dissolved indicator to various types of cell preparations. Endogenous, ubiquitous esterases cleave ester bonds and “trap” the now membrane impermeant Ca^2+^ sensitive dye intracellularly. The Ca^2+^ dependent amount of light emitted from these cells is generally measured using fluorescence microscopy, fluorescence microplate assays, or flow cytometry in combination with photon detection. The pharmacological and biophysical properties of these organic dyes have been reviewed (Paredes et al., 2008). One disadvantage of using these synthetic dyes is that they label tissue indiscriminately. For example, if you wish to study astrocytes in the CNS, application of the dye to the tissue will also label neighboring neurons. Further, many synthetic organic Ca^2+^ probes distribute into the cytosol, mitochondria, and other organelles making the measurements of “intracellular” Ca^2+^ more difficult to interpret.

During the same time period as synthetic dyes were improving, attempts to develop a genetically encoded [Ca^2+^]_i_ sensor were being performed. One such attempt has its origins in the use of complementary DNA from the *Aequorea victoria* green fluorescent protein (GFP) gene (Chalfie et al., 1994). GFP is a ∼27 kD protein that emits photons that fall within the visible spectrum when expressed in prokaryotic or eukaryotic cells upon proper excitation. GFP expression has become routine and widely used to examine an extensive range of biological questions ranging from gene expression to protein localization in living organisms. Over the past 20 years, GFP has been a major foundation for “directed evolution” into hundreds if not thousands of variations of the original wtGFP, many of which are currently being used as tools in fluorescent microscopy (Datta and Patterson, 2012). One genetic variant that developed about 7 years after GFP’s initial cloning was designed by Nakai et al. (2001). This research group genetically engineered a chimeric protein termed G“CaM”P as it was created from a fusion of circularly permutated GFP, calmodulin (CaM), and M13, a peptide sequence from myosin light chain kinase. Upon elevation of intracellular Ca^2+^, a conformational change occurred in GCaMP, enhancing fluorescent emission. Not surprisingly, due to the initial success of GCaMP as a Ca^2+^ sensor, it has been subsequently modified into increasingly higher numerical variants. More recent genetic versions of GCaMP are currently improving the signal-to-noise ratio of the fluorescence indicator, show improved kinetic responses, have variable Ca^2+^ binding affinities and other biophysical attributes that provide great flexibility in detection capacity (Akerboom et al., 2012; Sun et al., 2013). Additionally, “red shifted” genetically encoded calcium sensors have been generated that increase the spectral flexibility for imaging [Ca^2+^]_i_ (Yamada and Mikoshiba, 2012).

## GENETICALLY BASED EXPRESSION OF Ca^2+^ SENSORS

The combination of the mouse genetic strategies described above and the use of improved GCaMPs to monitor [Ca^2+^]_i_ in different cell types has been accomplished (Fletcher et al., 2009; Chen et al., 2012; Zariwala et al., 2012). These studies and others report in genetically identifiable cell types, changes in [Ca^2+^]_i_ with enhanced fluorescence as a function of various stimuli. Frequently in these studies, the increased signal in GCaMP fluorescence derives from the underlying mechanisms of neuronal action potentials and/or excitatory synaptic transmission. More broadly, the literature focuses on extracellular calcium entry as the source for increased cytosolic calcium signals. Among these mechanisms include the opening of voltage-gated calcium channels, NMDA-type glutamate receptors and calcium permeable AMPA-type glutamate receptors. The biophysical and pharmacological properties of evolving GCaMPs have improved to the detection level of single action potentials (Tian et al., 2009; Akerboom et al., 2012; Chen et al., 2013). This improving sensitivity has allowed investigators to correlate to a given rise in fluorescence with an accurate estimation of the number of action potentials while simultaneously detecting fluorescence in dozens of distinct cell types (Wachowiak et al., 2013).

However, what seems to be under-utilized by GCaMP functionality in the literature to date is the versatility to monitor increases in intracellular calcium from *extracellular independent* sources. As described above, there are critical sources of calcium which do not originate from the extracellular pool of calcium and contribute to microdomains of Ca^2+^ signaling (Berridge, 2006). It is now clear that cytosolic calcium signaling originating from extracellular or intracellular sources is capable of influencing different domains or compartments within a cell. The importance of these localized domains of Ca^2+^ is that they control distinct spatial actions in different regions of the cell. For example, the ER is an organelle whereby calcium is pumped against its natural concentration gradient by proteins like the sarco-/endoplasmic reticulum calcium ATPase (SERCA). Mitochondria are other vital intracellular organelles that can serve as critical sources of calcium upon proper stimulation. These two examples represent significant reservoirs of calcium that facilitate a local rise in [Ca^2+^]_i_ by a subcellular dependent fashion. As an example of the advancing technology integrating genetics and [Ca^2+^]_i_ imaging, Li et al. (2014) recently measured changes in Ca^2+^ from mitochondria ([Ca^2+^]_m_) in astrocytes using improved and compartmentalized GCaMP probes while Bengtson et al. (2010) monitored calcium changes within the nucleus of CA1 pyramidal neurons. While these studies used more traditional DNA vector transfection or viral infection methods to introduce the designed GCaMP into selected cell types, it highlights that mitochondrial (Rizzuto et al., 1995) or nuclear localization signal sequences can be added to the GCaMP sensors to direct the sensor to subcellular organelles or compartments. Other examples of clever genetic manipulations include membrane tethering sequences fused in frame as done with MARCKS-GCaMP2 (Mao et al., 2008) or Lck-GCaMP3 (Shigetomi et al., 2010). These latter two examples could be important starting points to more rigorously screen G_q/11_ calcium mobilization systems.

One advantage of the genetic techniques described above is that endogenous DNA recombination does a great deal of the work for the investigators without any requirements for survival surgery based methods. However, more invasive techniques, including stereotactic viral delivery or *in utero* electroporation (Yamada and Mikoshiba, 2012) can be used to extend the biological question posed.

## MONITORING ENDOGENOUS G_q/11_-COUPLED METABOTROPIC GLUTAMATE RECEPTORS USING ACUTE BRAIN SLICE PREPARATIONS

L-glutamate is the key excitatory neurotransmitter at the majority of synapses in the mammalian CNS. The initial detection of a distinct “metabolic” neuromodulatory glutamate receptor capable of generating IP_3_ occurred almost three decades ago (Nicoletti et al., 1986). It was also discovered during this time period that activation of unique glutamate receptors could elevate intracellular Ca^2+^ in a “spike like” fashion upon receptor stimulation in the absence of extracellular Ca^2+^ (Murphy and Miller, 1988). The cloning era was able to make great strides in our understanding of the glutamate receptor family structure and function. Two main divisions of L-glutamate binding proteins include the ionotropic (AMPA, NMDA, and kainate) and metabotropic glutamate receptors (mGluRs). Of the eight mGluRs, it is now apparent that Group I mGluRs: mGlu1 and mGlu5 preferentially couple to the synthesis of DAG and IP_3_ via G_q/11_. The widespread yet tissue specific expression of group I mGluRs suggests that these modulatory receptors have the ability to affect various functions in the CNS. Since their detection, mGluRs have been a focal point of various therapeutic aims to assist in alleviating symptoms of disease states ranging from Parkinson’s disease, diabetic neuropathy, melanoma, Autism spectrum disorders and generalized anxiety disorder (Niswender and Conn, 2010). For these reasons, GCaMP monitoring of G_q/11_-mediated rises in cytosolic calcium can deepen our knowledge of a vital receptor class.

**FIGURE 3:**
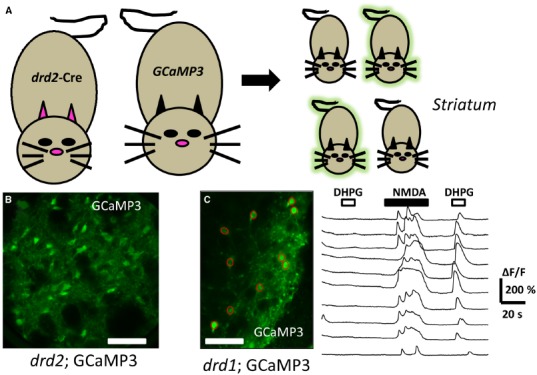
**Directed expression of genetically encoded calcium sensors to detect G_q/11_-GPCR activation. (A)** Mating a “*driver*” mouse directed to express cre in a selective dopamine D2 (*drd2*) fashion to a cre-dependent “*reporter*” GCaMP 3 mouse yields offspring which may inherit the driver-reporter combination of genes (*green* offspring). The offspring which do not inherit this combination will not express the calcium detector (*non-fluorescent* offspring). **(B)** Illustrates a confocal image of a coronal mouse brain section from an animal inheriting the *drd2*; cre and *rosa26*; GCaMP3 genes, permitting fluorescent detection of calcium changes in striatal dopamine D2 cells. **(C)** Live confocal image of an acute striatal brain slice showing *drd1*: GCaMP3 expressing neurons (green, *left*) with superimposed regions of interest (ROI, *red circles*) allowing genetically targeted calcium imaging. To the *right* are shown the time course of changes in [Ca^2+^]_i_ (ΔF/F) in the ROIs corresponding to cell bodies. Each trace represents a different ROI or cell body. The top horizontal bars above the traces represent the time duration that the drugs DHPG (10 μM, *open bars*), or NMDA (20 μM, *filled bars*) were acutely applied. Note the ability to detect GPCR activation following activation of the cells. Scale bars in **(B)** and **(C)**: 100 μm.

The Group I mGluRs are currently endowed with a rich array of pharmacological tools to dissect out particular branches and their role in signaling (Conn et al., 2014; Rook et al., 2015). One frequently used tool includes the compound (*S*)-3,5-dihydroxy-phenylglycine (DHPG). DHPG has been utilized as one of the most selective Group I mGluR orthosteric agonists. Recently, brain slice preparations of the striatum detected rises in [Ca^2+^]_i_ from genetically identified neurons following acute DHPG application using either organic dye loading methods (Chen et al., 2011; Plotkin et al., 2013) or GCaMP3 transgene expression (Partridge et al., 2014). In the latter case, confocal imaging combined with mouse genetics using dopamine D1 (*drd1*) or D2 (*drd2*) gene driven Cre recombinase provided a scaffold to monitor DHPG mediated changes in [Ca^2+^]_i_. The acute application of DHPG did not affect the basal fluorescence of GCaMP3 in most of the imaged D1^+^ or D2^+^ striatal neurons (Figure 3). However, in cells which were depolarized by either chemical or electrical means, a robust Ca^2+^ signal resulted when slices were acutely exposed to the G_q/11_-coupled GPCR agonist. These events were blocked by pretreatment with allosteric antagonists acting at mGluR1 and mGluR5. Further, the DHPG-mediated increase in GCaMP3 fluorescence was blocked by thapsigargin pre-treatment, an inhibitor of SERCA, strongly supporting a role for an intracellular source of calcium. The DHPG-mediated activation of native mGluRs as detected by GCaMP3, was fast and exhibited desensitization in the continued presence of this agonist. Further, in simultaneously current-clamped and GCaMP labeled neurons, the DHPG- mediated enhanced fluorescent signal was not associated with a change in membrane potential. This strongly supports the feasibility of these methods to detect active, endogenous GPCRs with GCaMP in an action-potential independent fashion.

Together, the data from that study indicate that striatal D1^+^ and D2^+^ projection neurons in acute brain slices express G_q/11_-coupled mGluRs that can be observed with good time resolution by calcium sensors. The ability to detect increases in GCaMP3 fluorescence was clearly enhanced by presumably “pre-filling” the intracellular stores with calcium. However, this combination of methods can clearly be useful to monitor dozens of distinct neurons simultaneously while probing the native state of receptors with pharmacological tools.

Within that same study, the flexibility of the method was shown as GCaMP3 expression was directed to more sparse interneurons by crossing somatostatin (*sst*; Taniguchi et al., 2011) or tyrosine hydroxylase (*th*; Lindeberg et al., 2004) gene-driven Cre recombination. In these striatal GABAergic interneuron subtypes, DHPG application produced robust increases in GCaMP3 fluorescence that differed significantly in the duration of fluorescent signal compared to those elicited in the *drd1* or *drd2* driven strains. Electrical recordings from the various GCaMP3 expressing interneuron subtypes indicated that DHPG did evoke action potentials in the two interneuron populations in this brain region. A recent study utilizing uncaging of IP_3_ came to a similar conclusion (Clements et al., 2013). Taken together, the data suggest a more classical type of G_q/11_-mediated change in intracellular calcium in projection type *drd1* or *drd2* expressing neurons. In contrast, the actions of DHPG acting upon interneuron populations could be utilizing the ability of G_q/11_ to couple to various TRP type channels (Gee et al., 2003; Ramsey et al., 2006). TRP channels were initially found to mediate photo-transduction in fruit flies and are non-selection cation channels. The open probability of several types of TRP channels can be enhanced upon activation of G_q/11_-coupled GPCRs. While more pharmacological evidence is necessary to validate this alternate pathway in striatal interneurons, this highlights the importance of the interpretation of the data. These studies and certainly others represent multidisciplinary techniques with rapidly evolving tools in which GPCRs can be assayed in natural states with relatively high temporal precision. This can greatly contribute to a deeper understanding of GPCR pharmacology while investigating the enormous heterogeneity of CNS cell types.

## CONCLUDING REMARKS AND FUTURE DIRECTIONS

G protein-coupled receptor signaling is a fundamental membrane-bound mechanism to detect selective changes in the local environment of animal cells. Because of the universal instrumentation of GPCRs across the animal kingdom, it is essential to understand the basic mechanisms on which they operate in an endogenous environment. Despite tremendous progress in our understanding of GPCR physiology and pharmacology, wide gaps remain in bridging the use of molecules that target these pathways to alleviate symptoms of disease and to develop clinically useful therapeutics. While this review has focused on one branch of the GPCR superfamily signaling pathway, opportunities to explore other canonical pathways like cAMP generation are being developed with luciferase based methods (Binkowski et al., 2011; DiRaddo et al., 2014). However, fluorescent protein-based cAMP indicators have lagged behind Ca^2+^ sensors and require further development with improved dynamic range and brightness.

Another goal moving forward in the GPCR field is to develop a “universal” detector of endogenous GPCR activation. The detection of protein–protein interactions (e.g., receptor-G-proteins, liberation of G_βγ_) would be one requirement of such a sensor. In fact, GPCR activation has been observed with several imaging probe techniques including intramolecular and intermolecular Förster resonance energy transfer (FRET)-based genetically encoded indicators (Lohse et al., 2012). However, an apparent constraint of this technique is that the introduction of dual fluorescent proteins (i.e., one acceptor and one donor), likely introduces steric hindrance and obstruction of essential protein–protein interactions necessary for energy transfer and the study of GPCRs in their endogenous states (Partridge et al., 2006). By combining the fields of fluorescent microscopy, mouse genetics and pharmacology we can enhance our understanding of GPCRs in their native state. Unanswered questions like the formation of various GPCR heteromers, altered pharmacology of heteromic receptors and cellular specificity can be answered with clever combinations of the methods mentioned in this review.

Improving GCaMP fluorescent signals have been detected using *in vivo* preparations (Hinckley and Pfaff, 2013; Dana et al., 2014), even in subcortical areas like the striatum (Cui et al., 2013). These elegant studies relied on the firing of action potentials to infer neuronal signaling. The challenge moving forward is to utilize fluorescent signals originating from the activation of GPCRs *in vivo*. This appears to be a reasonable challenge moving forward as detection of small but reliable Ca^2+^ increases can be detected in very fine mouse astrocyte processes both *in vitro* (Shigetomi et al., 2013) and *in vivo* (Otsu et al., 2015). These observations represent examples of calcium mobilization processes dependent upon acute GPCR activation.

### Conflict of Interest Statement

The author declares that the research was conducted in the absence of any commercial or financial relationships that could be construed as a potential conflict of interest.
